# Mental disorders and their comorbidities among children and adolescents aged 6 to 18 years in Lorestan Province

**DOI:** 10.22037/ijcn.v16i1.24465

**Published:** 2022-01-01

**Authors:** Hedayat NAZARI, Parvin SAFAVI, Zahra HOOSHYARI, Hosien PARSAMEHR, Farzaneh ABBASI MOTLAGH, Ameneh TAJIPOOR, Zohreh GOODARZI, Saba Shokri MOGHADAM, Shirin KUMASI JODAKI, Hamzeh SALEHI KIA, Maryam VEYSKARAMI, Samira BEYRANVAND

**Affiliations:** 1 Social Determinants of Health Research Center, Lorestan University of Medical Sciences, Khoramabad, Iran; 2 Psychiatry and Psychology Research Center, Roozbeh Hospital, Tehran University of Medical Sciences, Tehran, Iran; 3 Clinical Research Development Unit, Hajar Hospital, Shahrekord University of Medical Sciences, Shahrekord, Iran.; 4 Psychology and Educational science department, Allameh Tabataba’i University , Tehran, Iran.

**Keywords:** Mental disorders, psychiatric disorders, epidemiology, children, adolescents, prevalence

## Abstract

**Objectives:**

** The **profile of mental disorders has been changing over time. The aim of this study was to investigate the prevalence of mental disorders in children and adolescents in the Lorestan province of Iran.

**Materials & Methods:**

This community-based cross-sectional study was done on 1001 children and adolescents aged 6-18 years from Lorestan province randomly selected by multistage cluster sampling method. Children and their parents were interviewed using the Kiddie-Sads-Present and Lifetime Version (K-SADS-PL). Also, the comorbidities of psychiatric disorders were evaluated.

**Results:**

According to obtained results, 25.1% of participants were diagnosed to have at least one psychiatric disorder. The prevalence of psychiatric disorders was not significantly different between the two sexes (OR=0.876, P=0.378) and three age groups (P>0.05). this prevalence was significantly higher in rural areas than in urban areas (OR= 1.997, P=0) and was significantly lower in children of fathers with a high school diploma or higher education compared to children of less-educated fathers (P<0.05). On contrary, it was significantly more prevalent in children of mothers with high school and bachelor's degrees compared to illiterate mothers. (p<0.05). The most prevalent psychiatric disorders were oppositional defiant disorder (ODD) (5.9%), separation anxiety disorder (5.3%), and depressive disorders (5%). The most prevalent groups of psychiatric disorders included anxiety disorders (11.2%), behavioral disorders (9.4%), and mood disorders (5%). Behavioral disorders were highly comorbid with anxiety disorders (20.7%).

**Conclusion:**

Mental disorders affect a high proportion of children and adolescents in Lorestan province. There is a need for psychiatric facilities to provide for the needs of families to child mental health services.

## Introduction

The profile of psychiatric and mental disorders is changing over time. Hence, epidemiological studies are needed to know the current state of psychiatric disorders in children and adolescents and to schedule future services and resources for the modification of needs. Furthermore, current mental health problems in children and adolescents increase the risk of major psychiatric disorders and even suicide in adulthood (1). Limited studies have been conducted on the prevalence of psychiatric disorders in children and adolescents of Lorestan province, Iran. These studies have mainly assessed the prevalence of attention-deficit hyperactivity disorder (ADHD) and disruptive behavior disorders (DBDs) in children and adolescents of Khorramabad, the capital of the province. 

In a study on the prevalence of behavioral disorders among preschool children, it was indicated that of a total of 1977 cases, attention problem was the most common disorder among boys (6.1%), while emotional problems were the most common problem among preschool girls (17.2%) (2). The results of a cross-sectional study on 1200 primary school students in Khorramabad, using a questionnaire for parents and teachers, showed a prevalence of 6.5% for ADHD (3). Another study on 945 primary school students using standard questionnaires reported the prevalence of 17.3% for ADHD (4).

All these studies have used standardized questionnaires for screening specific psychiatric disorders and have been carried out in the setting of schools of Khorramabad, the main city of the province; but no epidemiological studies are using structured interviews assessing all psychiatric and mental disorders in children and adolescents of the province, including rural areas. This is the first epidemiological study conducted in Lorestan province addressing the prevalence of different psychiatric disorders in children and adolescents. The aim of the present study was to study the epidemiology of psychiatric and mental disorders in children aged 6 to 18 years in urban and rural areas of Lorestan province during 2015-2016. 

## Materials & methods

**Study Design**

This survey was an analytical cross-sectional study and a part of the Epidemiology of Psychiatric Disorders in Iranian Children and Adolescents (IRCAP), which was performed in Lorestan Province. Details of the study design and procedure can be found in the protocol article published by Mohammadi et al. (5). 

**Sampling**

A total of 1001 children and adolescents aged 6-18 years were selected randomly from Khoramabad, Lorestan, and some rural areas of the province by multistage cluster sampling method. One hundred and seventy cluster heads based on gender (boy/girl) and age (6-9 years, 10-14 years, and 15-18 years) blocks (6 samples in each) were randomly selected according to postal code. Cluster head selection from urban and rural areas was proportional to population. 

**Inclusion and Exclusion Criteria**

Inclusion criteria were being a citizen of Lorestan province (at least one-year residence in the Lorestan province), and age range of 6 to18 years. The exclusion criterion was children and adolescents with severe physical illness. 

**Data Collection**

The clinical psychologists participating in this study were instructed to complete the Persian version of Kiddie-Sads-Present and Lifetime Version (K-SADS-PL). Two trained psychologists referred to each selected home and interviewed the children and parents. The parents were interviewed to complete the screening questionnaires simultaneously and independently, and also the youths aged 11 years or older were interviewed to complete the questionnaires. The time required to complete the K-SADS was about 30 to 40 minutes. In addition, demographic data, including gender, age, education, parents’ education, and economic status were obtained. The interview started with questions about basic demographics. Information about presenting complaints and prior psychiatric problems was also obtained (6).

Kiddie-SADS-Present and Lifetime Version (K-SADS-PL): 

KSADS- PL, the Schedule for Affective Disorders and Schizophrenia for School-Age Children/Present and Lifetime Version, is a semi-structured psychiatric interview based on DSM-IV criteria. It contains five diagnostic groups: (a) affective disorders, including depression disorders (major depression and dysthymia), mania, and hypomania; (b) psychotic disorders; (c) anxiety disorders, including social phobia, agoraphobia, specific phobia, obsessive-compulsive disorder, separation anxiety disorder, generalized anxiety disorder, panic disorder, and posttraumatic stress disorder; (d) disruptive behavioral disorders, including ADHD, conduct disorder, oppositional defiant disorder; and (e) substance abuse, tic disorders, eating disorders, and elimination disorders (enuresis/encopresis) (7). 

Ghanizadeh et al. reported the reliability of the Persian version of this questionnaire to be 0.81, and the inter-rater reliability using test-retest was 0.69. The sensitivity and specificity of the Persian version of K-SADS were high (8). In a study by Polanczyk et al., the kappa coefficient was reported 0.93 (p<0.001) for affective disorders, 0.9 (p<0.001) for anxiety disorders, and 0.94 (p<0.001) for ADHD and disruptive behavior disorders (9). 

## Results

We used descriptive analysis and a 95% confidence interval to determine the frequency of psychiatric disorders in children and adolescents. Logistic regression was used to calculate the odds ratios and test the significant differences of prevalence according to demographic variables as well as to control the confounding variables.

A total of 1001 children and adolescents (49.2% boys and 50.8% girls) were included in the analysis, of whom 25.1% were diagnosed with psychiatric disorders. The frequency of demographic variables in the samples and the prevalence of psychiatric disorders in terms of these variables are shown in Table 1. 

Table 2 shows the significance of the relationship between demographic variables and psychiatric disorders in the samples. The results showed that gender (p=0.025, 95%CI =.485-.952), and father’s unemployment (p=0.016, 95%CI =1.262-9.620) had a significant correlation with the frequency of psychiatric disorders.

**Figure 1:**
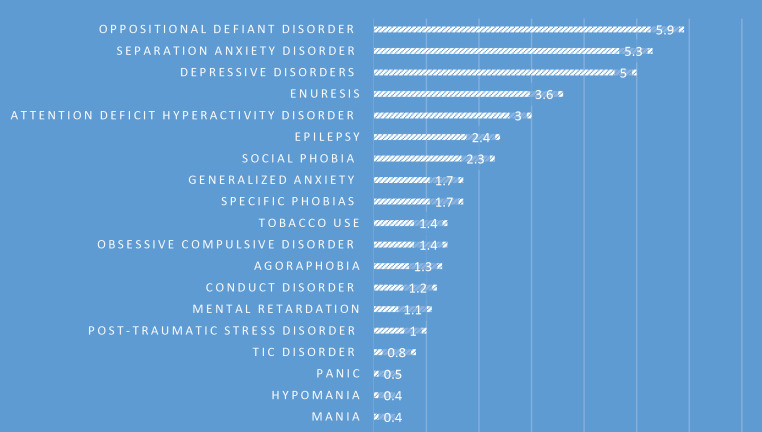
Prevalence of psychiatric disorders in children and adolescents of Lorestan Province

**Figure 2:**
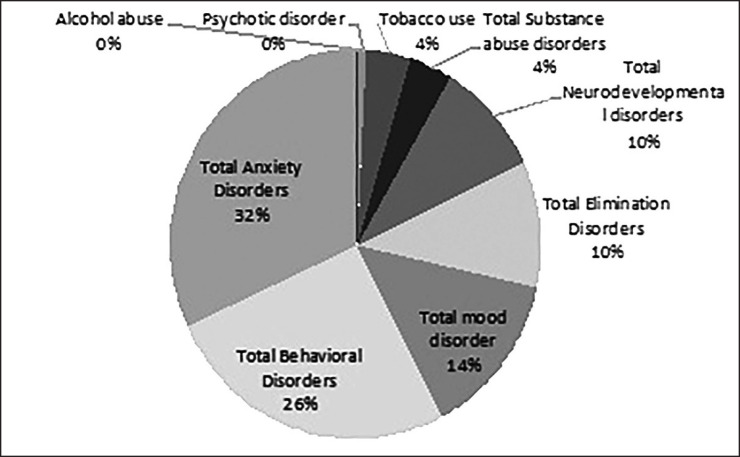
Prevalence of psychiatric disorders in children and adolescents of Lorestan Province

The prevalence rate of different types of psychiatric disorders is shown in Table 3. The prevalence of specific psychiatric disorders and classes of disorders are shown in Figures 1 and 2, respectively. Table 4 shows comorbidity disorders according to the type of psychiatric disorders in the province.

**Table 1:** Prevalence of Psychiatric and Mental Disorders in Terms of Demographic Variables in Children and Adolescents (6-18 years) of Lorestan Province

CI (95%)	**Psychiatric and Mental Disorders**		Total			
	p	n	P	N		
22.92-30.71	26.6	131	49.2	492	Boy	Sex
20.1-27.46	23.6	120	50.8	509	Girl	
22.28-31.52	26.6	93	34.9	349	6-9	Age (year)
18.91-27.86	23.1	78	33.8	338	10-14	
20.98-30.58	25.5	80	31.4	314	15-18	
18.68-24.49	21.4	164	76.4	765	Urban	Place of residence
30.96-43.18	36.9	87	23.6	236	Rural	
25.32-50.97	37.3	19	5.2	51	Illiterate	Father’s education
23.07-38.32	30.1	41	13.8	136	primary school	
24.84-37.16	30.7	65	21.5	212	Guidance & high school	
18.43-28.01	22.9	67	29.7	293	Diploma	
16.16-26.78	21	47	22.7	224	Bachelor’s degree	
10.09-27.61	17.1	12	7.1	70	Master’s or higher degrees	
			-	15	Missed	
12.84-37	22.7	10	4.5	44	Illiterate	Mother’s educations
20.51-33.89	26.7	44	16.7	165	primary school	
25.72-37.74	31.4	71	22.9	226	Guidance & high school	
17.54-26.35	21.6	72	33.8	333	Diploma	
19.51-32.05	25.3	46	18.5	182	Bachelor’s degree	
10-35	19.4	7	3.7	36	Master’s or higher degrees	
		1	-	15	Missed	
16.93-25.22	20.8	76	37.1	366	Public sector	Father’s job
24.4-31.82	28	156	56.6	558	Private sector	
20.59-42.97	30.6	19	6.3	62	unemployed	
			-	15	Missed	
18.5-34.42	25.7	29	11.4	113	Public sector	Mother’s job
19.42-53.78	34.6	9	2.6	26	Private sector	
22.29-28.11	25.1	213	85.9	849	unemployed (Housewife)	
			-	13	Missed	
22.48-27.85	25.1	251	100	1001	Total	

**Table 2:** Odds Ratios (95% CI) for total psychiatric and mental disorder in terms of demographic variables

**Variables and their categories**			**OR (crude)**	**CI (95%)**	**P-value**	**OR (adjusted)**	**CI (95%)**	**P-value**
Demographic variables	Sex	Male	1.00 Baseline					
		Female	.089	.011-.688	.020	.876	.652-1.177	.378
	Age group (year)	6-9	1.00 Baseline					
		10-14	2.030	.183-22.497	.564	.806	.561-1.157	.242
		15-18	10.013	1.261-79.498	.029	.928	.641-1.344	.692
	place of residence	Urban	1.00 Baseline					
		Rural	3.288	1.050-10.293	.041	1.997	1.399-2.848	0
	Father’s education	Illiterate	1.00 Baseline					
		primary school	.727	.370-1.428	.355	.610	.292-1.273	.188
		High school	.745	.393-1.410	.365	.600	.286-1.259	.177
		Diploma	.499	.266-.937	.031	.411	.187-.905	.027
		Bachelor’s degree	.447	0233-.859	.016	.350	.145-.846	.020
		Master’s or higher degrees	.348	.150-.809	.014	.241	.079-.731	.021
	Mother’s education	Illiterate	1.00 Baseline					
		primary school	1.236	.564-2.711	.596	1.809	.779-4.203	.168
		High school	1.557	.729-3.327	.253	2.831	1.195-6.708	.018
		Diploma	.938	.442-1.989	.867	2.237	.914-5.474	.078
		Bachelor’s degree	1.150	0527-2.509	.726	3.200	1.162-8.814	.024
		Master’s or higher degrees	.821	.277-2.430	.721	2.480	.634-9.699	.192
	Father’s job	Public sector	1.00 Baseline					
		Private sector	1.481	1.083-2.025	.014	1.150	.774-1.709	.490
		unemployed	1.686	.929-3.060	.086	1.149	.576-2.292	.694
	Mother’s job	Public sector	1.00 Baseline					
		Private sector	1.533	.616-3.816	.358	1.400	.516-3.797	.509
		Unemployed (Housewife)	.970	.619-1.521	.895	.638	.340-1.194	.160

**Table 3:** Prevalence of Psychiatric and Mental Disorders in children and adolescents (6-18 years) of the Lorestan province (6-18 years)

Psychiatric Disorders		Number	Percent	CI (95%)
Mood disorders	Depressive Disorders	50	5	3.81-6.53
	Mania	4	.4	0.16-1.02
	Hypomania	4	.4	0.16-1.02
	Total mood disorder	50	5	3.81-6.53
Psychotic disorder		2	.2	0.05-0.7
Anxiety Disorders	panic disorder	5	.5	0.2-1.16
	Separation Anxiety Disorder	53	5.3	4.07-6.86
	Social Phobia	23	2.3	1.54-3.43
	Specific Phobias	17	1.7	1.06-2.7
	Agoraphobia	13	1.3	0.76-2.21
	Generalized Anxiety	17	1.7	1.06-2.7
	Obsessive Compulsive Disorder	14	1.4	0.8-2.34
	Post-Traumatic Stress Disorder	10	1	0.5-1.83
	Total Anxiety Disorders	116	11.6	9.75-13.72
Behavioral Disorders	Attention-Deficit Hyperactivity Disorder	30	3	2.11-4.25
	Oppositional Defiant Disorder	59	5.9	4.59-7.52
	Conduct Disorder	12	1.2	0.7-2.09
	Tic Disorder	8	.8	0.4-1.57
	Total Behavioral Disorders	94	9.4	7.74-11.36
Neurodevelopmental disorders	Mental retardation	11	1.1	0.6-1.96
	Autism	3	.3	0.1-0.9
	Epilepsy	24	2.4	1.62-3.55
	Total Neurodevelopmental disorders	35	3.5	2.53-4.83
Substance abuse disorders	Tobacco use	14	1.4	0.84-2.34
	Alcohol abuse	1	0.1	0.02-0.56
	Total Substance abuse disorders	14	1.4	0.84-2.34
Elimination Disorders	Enuresis	36	3.6	2.61-4.94
	Encopresis	1	.1	0.02-0.56
	Total Elimination Disorders	37	3.7	2.7-5.06
Total		251	25.1	22.48-27.85

**Table 4:** Comorbidity disorders according to the type of psychiatric disorder in Lorestan Province

Elimination Disorders F(P)	Substance abuse disorders F(P)	Neurodevelopmental disorders F(P)	Behavioral Disorders F(P)	Anxiety Disorders F(P)	Mood Disorders F (P)	Comorbid disorder Main disorder
1(2)	1(2)	2(4)	9(18)	9(18)		Mood Disorders
9(7.8)	1(0.9)	6(5.2)	24(20.7)		9(7.8)	Anxiety Disorders
8(8.5)	3(3.2)	6(6.4)		24(25.5)	9(9.6)	Behavioral Disorders
3(8.6)	1(2.9)		6(17.1)	6(17.1)	2(5.7)	Neurodevelopmental disorders
0		1(7.1)	3(21.4)	1(7.1)	1(7.1)	Substance abuse disorders
	0	3(8.1)	8(21.6)	9(24.3)	1(2.7)	Elimination Disorders

## Discussion

The overall prevalence of mental and psychiatric disorders in the sample was 25.1%, which is higher than the worldwide prevalence of 13.4% for mental disorders in children based on the meta-analysis of Polanczyk et al. in 2015 (9). It is also higher than the prevalence rate (10.55%) of psychiatric disorders in the study by Mohammadi et al. on children and adolescents aged 6-18 years in five big provinces of Iran(11). Moreover, the prevalence of psychiatric disorders in Tabriz (15.69%) was the highest among other provinces and in Isfahan (6.2%) had the lowest rate. In a study on 879 subjects from Tehran interviewed based on DSM-IV criteria, the prevalence of psychiatric disorders, epilepsy, and mental retardation was estimated to be 21.5% (10). However, the prevalence in our study was lower than the study performed by Mowlavi *et al.* in Ardabil province (11). Farhoudian *et al.* (2007) showed that even though the pooled prevalence rates of psychiatric disorders in Iran are comparable to the rates in many other countries, these rates vary among different communities. This diversity was not suggested to be attributed solely to the different time frames and geographical locations of the studies. It might also be a result of differences in methodologies (e.g., using different tools), study procedures, and study quality (12).

The results showed no difference in the overall prevalence of psychiatric disorders between boys and girls. In a study by Alavi et al. on the prevalence of psychiatric problems in children aged 6 to 11 years in Tehran, the overall prevalence was reported to be 17.9, and no significant difference was observed between the two genders; however, bedwetting in boys and anorexia nervosa in girls were reported more than the other sex (13).

The rate of psychiatric disorders in rural areas was estimated to be significantly higher compared to urban areas; but in other studies, the rates of most types of psychiatric disorders in urban areas are estimated to be higher compared to rural areas (14). However, a recent study showed that the difference was not statistically significant (15). Other factors related to the place of residence, such as poverty, unemployment, and lower socioeconomic status should also be considered (16). 

The results also showed that psychiatric disorders were significantly less frequent in children of fathers with a high school diploma or higher education compared to children of less-educated fathers. On contrary, psychiatric disorders were significantly more prevalent in children of mothers with high school and bachelor's degrees compared to uneducated mothers. This finding requires the investigation of other sociodemographic factors related to parental education.

Psychiatric disorders were less prevalent in children of fathers working in the public sector compared to the private sector or unemployed fathers, but this difference was not significant.

The most prevalent psychiatric disorders in the study population were oppositional defiant disorder (ODD) (5.9%), separation anxiety disorder (5.3%), and depressive disorders (5%). In the study by Alavi et al. hyperactivity problems, oppositional defiant problems, and separation anxiety accounted for the highest frequency with 8.6%, 7.3%, and 5.9%, respectively (14). The prevalence of ADHD was 3% in the present study, which is lower than the estimate in previous studies (2, 3) because, in the present study, the interview was used for diagnosis, but the previous studies used standard screening questionnaires. Mohammadi et al., like our study, reported ODD (4.45%) with the highest prevalence. In addition, ADHD had the highest prevalence in boys (5.03%), and ODD had the highest prevalence in girls (4.05%). 

Enuresis was reported in 3.5% of the study population. In another study performed by Torkashvand et al. in Rafsanjan, the prevalence of nocturnal enuresis in school-aged children was nearly 10% (17). In a study on 710 children selected from the schools of Khoramabad, 8% of the children had nocturnal enuresis. The prevalence of nocturnal enuresis in the boys (10.7%) was higher compared to the girls (5.4%). In this study, only preschool and elementary school children were included (18).

The most prevalent groups of psychiatric disorders based on DSM IV classification were anxiety disorders (11.2%), behavioral disorders (9.4%), and mood disorders (5%). Based on a Meta-analysis, the worldwide prevalence of mental disorders was as follows: anxiety disorders: 6.5%, depressive disorders: 2.6%, major depression: 1.3%, ADHD: 3.4%, disruptive disorders: 5.7%, oppositional defiant disorder: 3.6%, and conduct disorder: 2.1%. A previous study (2005) performed on elementary-school students of Khorramabad showed that 21.4% of children had DBD (17.7% oppositional defiant disorder and 3.7% conduct disorder) (1). The high prevalence of DBDs may reflect the lack of behavior management skills in parents. 

Comorbidity was common in children with psychiatric disorders. The most prevalent comorbidity of anxiety disorders was behavior disorders (20.7%). The most prevalent comorbidity of mood disorders was anxiety disorders (18%) and behavioral disorders (18%), and behavior disorders were highly comorbid with other psychiatric disorders.

**InConclusion, **Psychiatric disorders affect a high proportion of children and adolescents in Lorestan province. There is a need for more public education and modification of psychiatric facilities to provide the needs of families and psychiatric services.

## Author’s Contribution

Mohammad Reza Mohammadi contributed to the study conception and design. Hedayat Nazari led monitoring and implementation of the study. All authores cooperated in executive steps of the study. The first draft of the manuscript was written by Parvin Safavi, and all authors commented on previous versions of the manuscript. All authors read and approved the final manuscript.

## Conflicts of interest

The authors have no conflicts of interest to declare that are relevant to the content of this article.
